# Dry Nutrition Delivery System Based on Defatted Soybean Particles and Its Application with β-Carotene

**DOI:** 10.3390/molecules28083429

**Published:** 2023-04-13

**Authors:** Chunyu Wu, Xuewen Ouyang, Xiaoya Zhou, Xiaofei Li, Hongbo Li, Wenying Li, Cuixiang Wan, Bo Yu, Sobhy El-Sohaimy, Zhihua Wu

**Affiliations:** 1 State Key Laboratory of Food Science and Technology, Nanchang University, Nanchang 330047, China; 2 College of Food Science and Technology, Nanchang University, Nanchang 330031, China; 3 Aistarfish Technology Co., Ltd., Hangzhou 310012, China; 4 Anhui Huaheng Biotechnology Co., Ltd., Hefei 230031, China; 5 Zhaotong Health Vocational College, Zhaotong 657000, China; 6 Sino-German Joint Research Institute, Nanchang University, Nanchang 330047, China; 7 Department of Technology and Organization of Public Catering, Institute of Sport, Tourism and Service, South Ural State University, 454080 Chelyabinsk, Russia

**Keywords:** dry, nutrition delivery systems, defatted soybean particles, β-carotene, stability, bioavailability

## Abstract

Many nutrition delivery systems (NDSs) have been developed for the encapsulation, protection, and delivery of bioactive compounds, such as β-carotene. Most of those systems were prepared in solution, which is inconvenient for transportation and storage in the food industry. In the present work, we constructed an environmentally friendly dry NDS based on defatted soybean particles (DSPs) by milling a β-carotene-DSP mixture. The loading efficiency of the NDS reached 89.0%, and the cumulative release rate decreased from 15.1% (free β-carotene) to 6.0% within 8 h. The stability of β-carotene in the dry NDS was found to have increased in a thermogravimetric analysis. Stored for 14 days at 55 °C or under UV irradiation, the retaining rates of β-carotene in the NDS increased to 50.7% and 63.6%, respectively, while they were 24.2% and 54.6% for the free samples. The bioavailability of β-carotene was improved by the NDS too. The apparent permeability coefficient of the NDS reached 1.37 × 10^−6^ cm/s, which is 12 times that of free β-carotene (0.11 × 10^−6^ cm/s). Besides being environmentally friendly, the dry NDS can facilitate carriage, transportation, or storage in the food industry, and similar to other NDSs, it improves the stability and bioavailability of nutrients.

## 1. Introduction

A major trend in the modern food industry has been the development of functional foods specifically designed to improve human health and wellbeing [1]. Food-sourced bioactive compounds, e.g., carotenoids, polyphenols, vitamins, and functional lipids, have drawn much attention due to their health benefits such as anti-oxidant, anti-cancer, anti-diabetes, and cardiovascular disease-preventing functions [2]. However, the poor solubility, low stability, and limited bioavailability of sensitive bioactive compounds greatly limited their application in the food industry. Therefore, various carriers were developed to improve their dispersibility, stability, and bioavailability [3]. Substantial interest now focuses on the development of nanoscale systems for the encapsulation, protection, and delivery of lipophilic nutrients, vitamins, and nutraceuticals, and nanoscale delivery systems have considerable potential within the food industry, which allows for an easier design of novel functional foods with desirable health benefits [1]. Delivery systems that are more environmentally friendly and more convenient and effective are needed in the food industry.

Challenges remain in ensuring that functional ingredients are sustained and remain ‘active’ and ‘bioavailable’ after food processing and storage [4]. For example, β-carotene, having the highest provitamin A activity, is essential to the human body by providing antioxidant and anti-inflammatory effects, improving the body’s immunity, and treating some diseases such as nyctalopia and xerophthalmia and is therefore a strong candidate for incorporation into functional foods [5,6,7]. Different types of delivery systems including Pickering emulsion, casein/gum tragacanth, nanoemulsions, bio-based nanoparticles, and cellulose were developed to overcome poor water solubility, crystallization, chemical instability, and/or low bioavailability [8,9,10,11,12,13].

Among these systems, emulsions are considered to be particularly suitable for the delivery of functional ingredients such as β-carotene into food systems because they are common food types and easy to add into different foods [14]. However, emulsions need oil–water interfaces, and the aqueous medium provides the necessary impetus for the formation of microemulsion droplets [15]. Thus, liquid emulsion delivery systems inevitably suffer from poor physical or chemical stability, and carriage, transportation, or storage inconvenience, which are commonly associated with liquid formulations in practical applications [16]. Although a solid self-emulsifying delivery system could make the delivery system solid [17], both the formulations and procedures of emulsion fabrication are pretty complicated [18,19].

A dry nutrition delivery system (NDS) is constructed without liquid, neither oil nor water, which not only is environmentally friendly but also facilitates carriage, transportation, or storage in the food industry. Here, a nutrition delivery system based on defatted soybean nano particles [20] was developed. Defatted soybean particles (DSPs) have been proved to be safe at a single dose of 10 g/kg body weight, regardless of the particle size, and they can be a potential carrier in nutrition delivery due to their aggregation behavior [21].

In the present work, a β-carotene delivery system was constructed based on DSPs without a solution. The NDS was easy to construct, and β-carotene was proved to be carried successfully.

## 2. Results and Discussion

The NDS was constructed, and its size and zeta potential are summarized in Table 1. The size of a DSP could reach around 100 nm when milled by the ball mill [21]. Here, DSPs with sizes of 600–700 nm were used to construct the NDS. After the DSPs were mixed with β-carotene, the hydrodynamic sizes of the particles increased. The size of the 10 min βc-DSP was 894.3 ± 96.7 nm, and 20 min more of milling decreased the size to 829.7 ± 29.0 nm. The center size decreased with milling time, and the distribution scale narrowed at the same time. The surface zeta potential of the samples changed a little after being mixed with β-carotene (from −14.84 ± 0.52 to −15.53 ± 0.54) and changed a little more after more milling. Furthermore, the diameters of the DSPs and NDS are at the submicron level. This size is safe for delivery [22,23], which was also proved by the cell model.

No liquid, either aqueous or organic solvents, was added during the NDS construction, which makes it a dry NDS. A dry NDS facilitates application in the food industry, and the process of construction is simple. Additionally, in terms of transportation or storage, the powder of a dry NDS is much more useful than a solution of traditional NDS. Moreover, a dry NDS is environmentally friendly. It need not be dried, saving on energy. It also produces no waste, such as an organic solvent, either.

**Figure 1:**
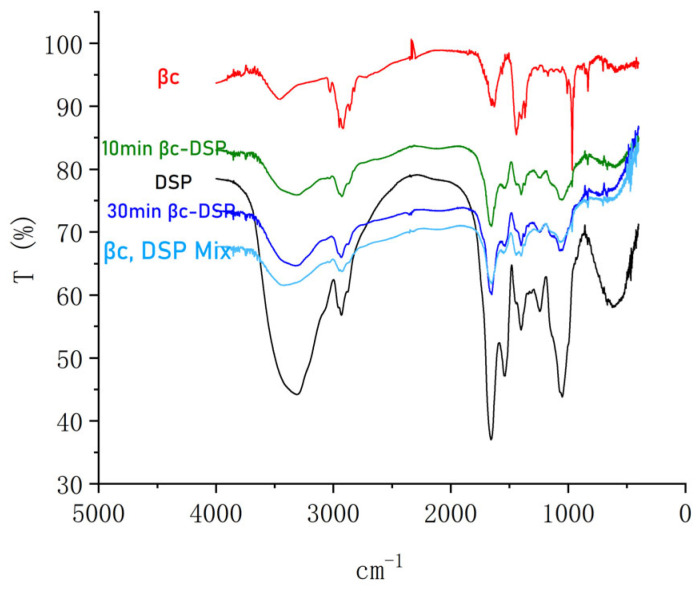
The infrared spectrum of the dry nutrition delivery system. βc: β-carotene; DSP: Defatted soybean particles; 10 min βc-DSP: ball mill again for 10 min; 30 min βc-DSP: ball mill again for 30 min.

The FT-IR spectra of β-carotene, DSPs and their physical mixture, and the NDS complexes are shown in Figure 1. In the β-carotene spectrum, the intense bands located at 3030 cm^−1^–2849 cm^−1^ were observed and identified as characteristics of the structural carbons of carotenoids (C=C stretching and aliphatic C-H stretching). The bands at 963–948 cm^−1^ and 827–652 cm^−1^ correspond to the C-C and methylene rocking vibrations [24]. In the DSP spectrum, the property of the protein is clear. The broad band at 3312 cm^−1^ indicates the stretching of single bonds -NH2 and -NH. At 1401.16 cm^−1^, the vibration -COO- was recorded [25,26]. In contrast, in β-carotene-DSP complexes, both 10 min βc-DSP and 30 min βc-DSP, the peak of 3312 cm^−1^ moved to 3453 cm^−1^, which indicated that there is a hydrogen bond.

The loading efficiency of the NDS decreased with milling time. In 10 min βc-DSP, it was 79.4% while it was 89.0% in 30 min βc-DSP. In the β-carotene-DSP mixture, the peak at 970–960 cm^−1^, characteristic of β-carotene, is strong. In the complexes, the peak remained clear in 10 min βc-DSP, but it disappeared in 30 min βc-DSP. This also proved that β-carotene was packaged in DSPs more tightly with more time. The loading efficiency was quite high compared with the other complexes [10,27,28,29]. Considering the safety of DSPs [21], the loading rate is sufficient for the food industry.

**Figure 2:**
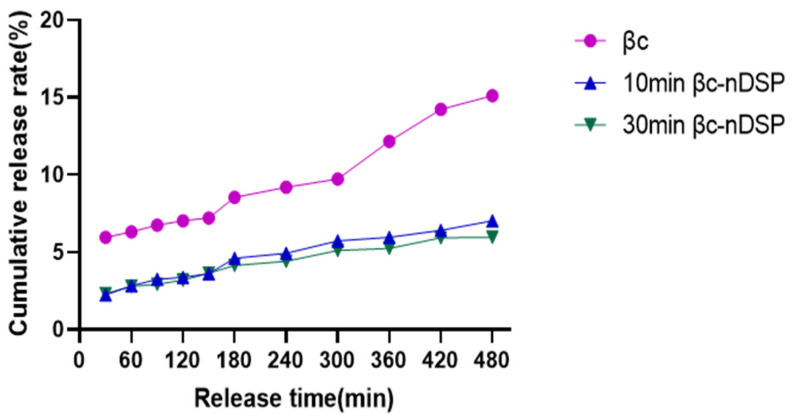
The cumulative release curves of β-carotene and the dry delivery system. βc: β-carotene; 10 min βc-DSP: ball mill again for 10 min; 30 min βc-DSP: ball mill again for 30 min.

The cumulative release rate of β-carotene is presented in Figure 2. The cumulative release rate of free β-carotene, 10 min βc-nDSP, and 30 min βc-nDSP were 15.1%, 7.0%, and 6.0% in 8 h, respectively. Release model fitting was performed on the release curves to compare the mass transport mechanism of the different samples [30]. The release of free β-carotene fitted the zero-order model, while in the complexes, the release from NDS better fitted the Higuchi model (see Supplementary Materials). These results also demonstrated that the dry nutrition delivery system was prepared successfully and slowed down the release of β-carotene.

In order to evaluate the stability of β-carotene in the dry NDS, a thermogravimetric analysis was carried out on the samples and how much β-carotene in solution was retained was investigated. Both results clearly indicated that the β-carotene in NDS showed relatively higher thermal stability as compared with that of the free sample [31,32].

**Figure 3:**
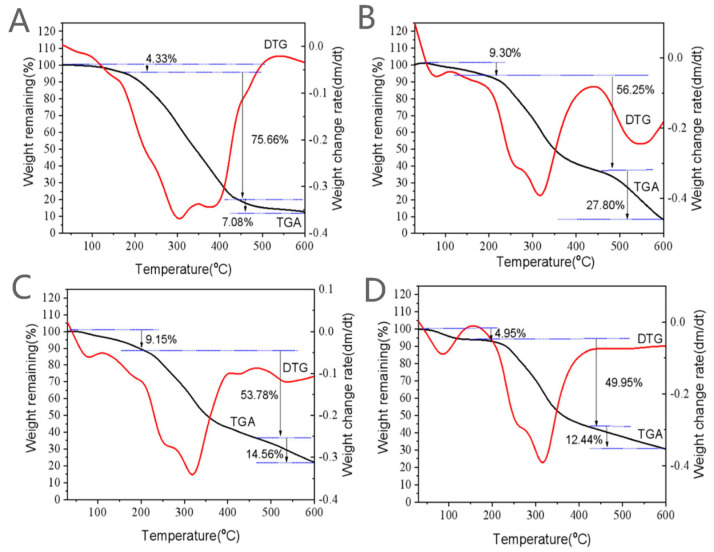
TGA-DTG curve of β-carotene and the nutrition delivery system. (**A**) β-carotene; (**B**) DSPs; (**C**) 10 min βc-DSP; (**D**) 30 min βc-DSP.

The thermogravimetric analysis (in Figure 3) showed that the weight loss rate of free β-carotene was divided into three stages with the change in temperature, and the most important thermal decomposition stage was the second stage, at 200–450 °C; the weight loss rate at this stage reached 75.7%. The change in weight loss for 10 min βc-DSP and 30 min βc-DSP with temperature were also divided into three stages, primarily decomposed at 230–448 °C in the second stage, and the weight loss rates were 53.8% and 50.0%, respectively.

**Figure 4:**
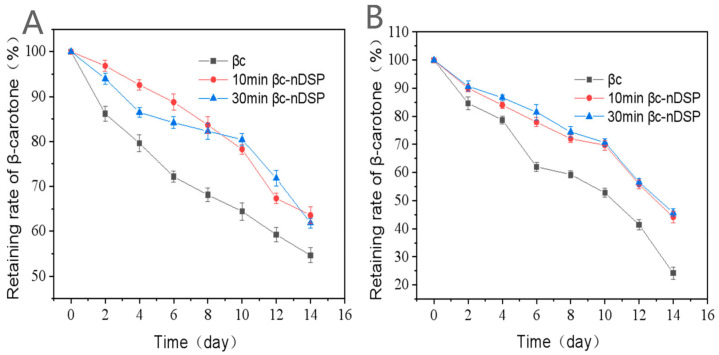
The storage stability of β-carotene in the nutrition delivery system. (**A**) Stored under UV irradiation; (**B**) stored at 55 °C. βc: β-carotene; 10 min βc-DSP: ball mill again for 10 min; 30 min βc-DSP: ball mill again for 30 min.

In solution, the retaining rates of free β-carotene, 10 min βc-DSP, and 30 min βc-DSP stored for 14 days under dark conditions at 55 °C were 24.2%, 44.1%, and 50.7%, respectively, while they were 54.6%, 63.6%, and 61.8%, respectively, under UV irradiation at room temperature. As shown in Figure 4, the retention of β-carotene in NDS is always higher than in the free one. The β-carotene in NDS is obviously stabler than the sample in solution [33,34].

**Figure 5:**
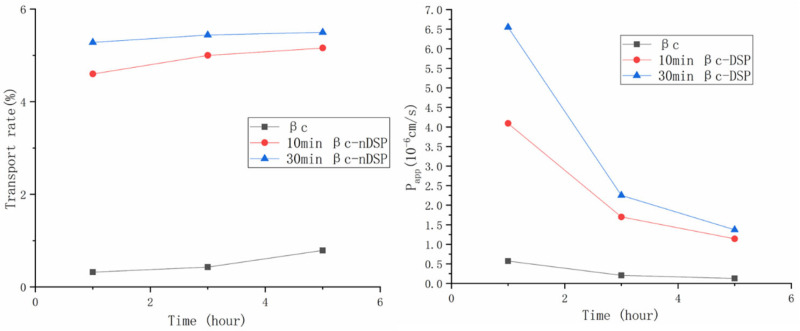
The transport rate and apparent permeability coefficient of β-carotene in NDS. βc: β-carotene; 10 min βc-DSP: ball mill again for 10 min; 30 min βc-DSP: ball mill again for 30 min.

The absorption of β-carotene was evaluated on a Caco-2 monolayer cell model (Figure 5). To perform a transportation study, Caco-2 cell monolayers were developed after the cytotoxicity evaluation [9,35,36]. No effect on the activity of the Caco-2 cells was found when the concentrations of β-carotene and its dry transport system were no more than 50 μg/mL. Within 5 h, the apparent permeability coefficient (Papp) and transport rate of 30 min βc-DSP were 1.37 × 10^−6^ cm/s and 5.5%, respectively, which were higher than the Papp and the transport rate of 10 min βc-DSP (Papp was 1.28 × 10^−6^ cm/s, and transport rate was 5.2%). While for free β-carotene, the Papp and transport rate were 0.11 × 10^−6^ cm/s and 0.8%, respectively, which were much lower than those of the complexes. Thus, the dry NDS can improve the bioavailability of β-carotene [3,9].

**Figure 6:**
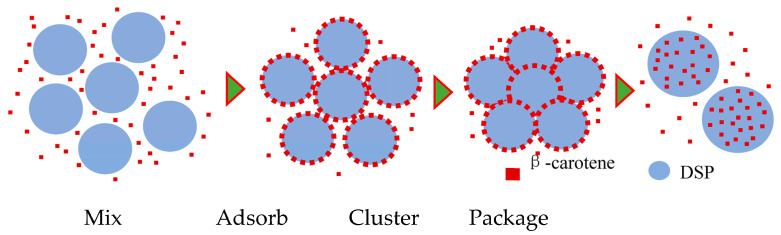
A schematic diagram of the dry nutrition delivery system construction. After the β-carotene mixes with DSPs, it will be adsorbed onto the surface. During DSP clustering and crushing, the β-carotene is packed inside. βc: β-carotene; DSP: Defatted soybean particles.

In the dry β-carotene delivery system, the aggregation of DSPs [21] contributes to the construction. The schematic diagram in Figure 6 describes the formation of a dry NDS. When mixed with DSPs, β-carotene is adsorbed onto the surface of the particles [37]. Combined with the aggregation of DSPs, those β-carotenes move into the cluster. The clusters are then broken during milling, and the β-carotene is packaged inside at the same time. Up to 89% β-carotene can be packaged into the NDS within 30 min.

## 3. Materials and Methods

### 3.1. NDS Construction

Defatted soybean particles (DSPs) were prepared following the method described by Wu et al. [20]. In brief, a commercially available defatted soybean meal was dried and milled by an FDV-SS ultra-fine crusher (Youqi Co., Taibei, China) and then subjected to a PULVERISETTE 6 high-energy planetary ball mill (FRITSCH, Idar-Oberstein, Germany) for 30 min so that the DSPs could be used in the nutrition delivery system (NDS). The β-carotene was mixed with the DSPs at a ratio of 1:3 (*w*/*w*) and subjected to the ball mill again for 10 min or 30 min to construct the NDS, named as 10 min βc-DSP and 30 min βc-DSP, respectively.

### 3.2. NDS Characterization

Particle characterization was performed following the methods of Tong et al. [21], and the DSPs and NDS were dispersed in distilled water at 0.5 mg/mL as a stock solution. These solutions were sonicated in a 650-92 Ultrasonic homogenizer (Biosafer, Hongkong, China) and diluted (if needed) before characterization. A laser particle size analyzer (Nano ZS90, Malvern, UK) was applied to determine the hydrodynamic sizes and surface zeta potential of the samples (0.1 mg/mL).

A Fourier transform infrared spectrometer (FT-IR) was used to collect the solid NDS in a platinum accessory and a diamond crystal was collected using a Fourier transform spectrometer (PerkinElmer, USA) following the methods of Afonso et al. [10]. The scanning range of the wave number (σ) was 500–4000 cm^−1^, the resolution was 2 cm^−1^, the scanning interval was 1 cm^−1^, and the number of cumulative scans was 4.

The amount of β-carotene loaded into the NDS was quantified by HPLC (LC-15C, Shimadzu, Kyoto, Japan) with a C18 column (250 mm × 4.6 mm, 5 μm, Shimadzu WondaSil C18 Superb) following the methods of Li et al. [38]. In brief, 1 mg of dry NDS was diluted in 10 mL of PBS (pH 7.4); then, 1 mL of hexane/ethanol (3:2, *v*/*v*) was added to extract the free β-carotene from the solution for quantification. The loading efficiency was calculated as follows:

(1) $$\mathrm{Loading}\;\mathrm{efficiency}=\left(1-\frac{\mathrm{weight}\;\mathrm{of}\;\mathrm{free}\;\beta -\mathrm{carotene}}{\mathrm{weight}\;\mathrm{of}\;\mathrm{total}\;\beta -\mathrm{carotene}}\right)\times 100\%$$

### 3.3. Cumulative Release and Model Fitting

The release kinetics of β-carotene from NDS were studied using the dialysis strategy [39]. The solution of NSD or free β-carotene (100 μg/mL, 10 mL) was added to pre-soaked dialysis bags with a molecular weight cutoff of 8 kDa. Then, these bags were sealed and incubated in 1 L pure water at 37 °C, 100 rpm. The quantity of released β-carotene was determined by HPLC at different intervals following the methods of Li et al. [38]; then, the release rate at each time point was calculated by *w*/*w*.

The release curve was fitted with different models, so as to describe the mass transport mechanism and to test if the β-carotene was carried [30]. These models are as follows:

Zero-order model:

(2) $$\frac{M_{t}}{M_{\infty }}=kt$$

First-order model:

(3) $$ln\left(1-\frac{M_{t}}{M_{\infty }}\right)=-kt$$

Higuchi model:

(4) $$\frac{M_{t}}{M_{\infty }}=k\sqrt{t}$$

Korsmeyer–Peppas Model:

(5) $$\frac{M_{t}}{M_{\infty }}=kt^{n}$$

where *M_t_* is the cumulative absolute amount of β-carotene released at time t; *M*_∞_ is the absolute cumulative amount of drug released at infinite time; *k* is a constant incorporating the property of NDS; and *n* is the release exponent, indicative of the mechanism of drug release.

### 3.4. Stability of NDS

Thermogravimetry (TGA) and differential thermogravimetry (DTG) analyses for the NDS were carried out on 4–6 mg samples using a SDT Q600 TG/DSC thermal analyzer (TA Instrument Corp., New Castle, DE, USA) at a heating rate of 10 °C/min from 30 to 600 °C with a constant nitrogen flow of 20 mL/min [39].

For stability during storage, free β-carotene and NDS with β-carotene were diluted in ultrapure water (0.1 mg/mL). Two groups of samples were stored, at 25 °C with UV or at 55 °C in the dark. The concentration of β-carotene was detected by a spectrophotometer (Thermo, Denver, CO, USA) at 450 nm each day for two weeks. The β-carotene retention rate was calculated using the following formula:

(6) $$R_{n}=\frac{OD_{n}}{OD_{0}}\times 100\%$$

where *R_n_* denotes β-carotene retention, *OD_n_* indicates the *OD* on n day, and *OD*_0_ represents the initial *OD* of β-carotene.

### 3.5. In Vitro Safety and Absorption Assessment

Caco-2 cells were purchased from the Cell Bank of Chinese Academy of Sciences (Shanghai, China) and maintained in Dulbecco’s modified Eagle medium (DMEM; Sigma-Aldrich, Bellefonte, PA, USA) at 37 °C under an atmosphere containing 5% CO_2_. The growth medium was changed daily in the first weeks, followed by replacement of the medium three times a week. The cells were subcultured weekly by trypsin-EDTA (Solarbio, Beijing, China) treatment [40,41]. Additionally, the effect of NDS on cell proliferation and the viability of Caco-2 cells were measured via a CCK-8 (Solarbio, Beijing, China) assay [41,42].

The transport of β-carotene loaded into an NDS across Caco-2 cell monolayers was studied following the method of Rosso et al. [19]. Caco-2 cells were seeded onto Transwell inserts (ThinCert™ Greiner Bio-One, Les Ulis, France) in 24-well tissue culture plates (Cellstar^®^ Greiner Bio-One, Les Ulis, France).

The cells were grown and differentiated so as to form a cell monolayer with transepithelial electrical resistance (TEER) values around 300 Ω·cm^2^. Then, transport experiments across the Caco-2 monolayers were carried out. After washing with HBSS, 0.5 mL of the free β-carotene or loaded β-carotene (both containing 25 μg/mL of β-carotene) was added at the apical compartment of the inserts (as the donor), while the basolateral compartment was filled with 1.5 mL of HBSS. After 1 h, 3 h and 5 h of incubation, the medium in the basolateral compartment was collected, and the β-carotene in the medium was extracted and quantified by HPLC.

The transport rate of β-carotene was calculated as

(7) $$Transport\;rate=\frac{C_{b}*1.5}{0.5*25}\times 100\%$$

where *C_b_* is the concentration of β-carotene in the basolateral compartment, μg/mL.

The apparent permeability (Papp) coefficient (expressed in cm/second) is calculated as follows:

(8) $$P_{app}=\frac{dQ}{dt\times A\times C}$$

where *dQ*/*dt* is the solute flux (μg/s) across the barrier, *A* is the surface area (1.12 cm^2^), and *C* is the initial donor concentration (μg/mL).

### 3.6. Statistical Methods

The data were expressed as means ± standard deviation (SD) for three replicates. One-way ANOVA was used to analyze the significant differences between the groups using GraphPad Prism software (La Jolla, Calif). Values of *p* < 0.05 were considered significant.

## 4. Conclusions

In the present work, a novel dry nutrition delivery system (NDS) was constructed based on defatted soybean particles. Because this NDS is liquid free, it is environmentally friendly and without waste. Additionally, it facilitates construction, transportation, and storage in the food industry. Similar to other NDSs, a dry NDS is effective and improves the stability and bioavailability of nutrients. The construction of a liquid free NDS is promising in the food industry due to its convenience and efficiency.

## Figures and Tables

**Table 1:** Size and zeta potential of the delivery system.

Samples	DSP	10 Min βc-DSP	30 Min βc-DSP
PS (nm)	665.4 ± 40.0	894.3 ± 96.7	829.7 ± 29.0
z (mV)	−14.84 ± 0.52	−15.53 ± 0.54	−15.20 ± 0.86

DSP: Defatted soybean particles; 10 min βc-DSP: ball mill again for 10 min; 30 min βc-DSP: ball mill again for 30 min.

## Data Availability

The data are contained within the article.

## Supplementary Materials

The following supporting information can be downloaded at: https://www.mdpi.com/article/10.3390/molecules28083429/s1, Table S1: The release model fitting of free β-carotene and in the delivery system.

## Sample Availability

Samples are available from the authors.
