# Supplementary material for: Dry Nutrition Delivery System Based on Defatted Soybean Particles and Its Application with β-Carotene
Source: Molecules. 2023 Apr 13;28(8):3429. doi: 10.3390/molecules28083429 (PMC10145488; doi:10.3390/molecules28083429)
Supplement: Supplementary file 1 [file molecules-28-03429-s001.zip › molecules-2277918-supplementary.pdf]

Supplementary material

Table S1 The release model fitting of free β-carotene and in the delivery system

		Equation	R^2
β-carotene	Zero order model	$M_t/M_\infty=4.68\ t+0.021$	0.96259
	First order model	$\ln(1-(-2.6\times10^{-5})\ M_t/M_\infty)=9.38\times10^{-7}\ t$	0.18659
	Higuchi model	$M_t/M_\infty=0.57\ t^{1/2}+1.36$	0.8869
	Korsmeyr-Peppas model	$M_t/M_\infty=0.88\ t^{0.44666}$	0.87067
10 min βc-DSP	Zero order model	$M_t/M_\infty=2.24\ t+0.010$	0.97162
	First order model	$\ln(1-(0.15)\ M_t/M_\infty)=-0.00631\ t$	0.89042
	Higuchi model	$M_t/M_\infty=0.29\ t^{1/2}+0.43$	0.98321
	Korsmeyr-Peppas model	$M_t/M_\infty=0.41\ t^{0.45486}$	0.97858
30 min βc-DSP	Zero order model	$M_t/M_\infty=2.31\ t+0.0084$	0.96999
	First order model	$\ln(1-(0.17)\ M_t/M_\infty)=-0.00782\ t$	0.83459
	Higuchi model	$M_t/M_\infty=0.24\ t^{1/2}+0.84$	0.98203
	Korsmeyr-Peppas model	$M_t/M_\infty=0.53\ t^{0.4941}$	0.97498
